# NK cells and solid tumors: therapeutic potential and persisting obstacles

**DOI:** 10.1186/s12943-022-01672-z

**Published:** 2022-11-01

**Authors:** Le Tong, Carlos Jiménez-Cortegana, Apple H.M. Tay, Stina Wickström, Lorenzo Galluzzi, Andreas Lundqvist

**Affiliations:** 1 Department of Oncology-Pathology, Karolinska Institute, Stockholm, Sweden; 2 Department of Radiation Oncology, Weill Cornell Medical College, New York, NY USA; 3 Department of Medical Biochemistry, Molecular Biology and Immunology, Faculty of Medicine, University of Seville, Seville, Spain; 4 Department of Biological Science, Nanyang Technological University, Singapore, Singapore; 5 Sandra and Edward Meyer Cancer Center, New York, NY USA; 6 Caryl and Israel Englander Institute for Precision Medicine, New York, NY USA

**Keywords:** Adoptive cell therapy, CAR T cells, CGAS/STING1 signaling, Immune checkpoint inhibitors, PD-L1, T_REG_ cells

## Abstract

Natural killer (NK) cells, which are innate lymphocytes endowed with potent cytotoxic activity, have recently attracted attention as potential anticancer therapeutics. While NK cells mediate encouraging responses in patients with leukemia, the therapeutic effects of NK cell infusion in patients with solid tumors are limited. Preclinical and clinical data suggest that the efficacy of NK cell infusion against solid malignancies is hampered by several factors including inadequate tumor infiltration and persistence/activation in the tumor microenvironment (TME). A number of metabolic features of the TME including hypoxia as well as elevated levels of adenosine, reactive oxygen species, and prostaglandins negatively affect NK cell activity. Moreover, cancer-associated fibroblasts, tumor-associated macrophages, myeloid-derived suppressor cells, and regulatory T cells actively suppress NK cell-dependent anticancer immunity. Here, we review the metabolic and cellular barriers that inhibit NK cells in solid neoplasms as we discuss potential strategies to circumvent such obstacles towards superior therapeutic activity.

## Introduction

Cancer immunotherapy has gained considerable momentum in the past decade, especially in the forms of immune checkpoint inhibition [1] and adoptive cell therapy (ACT), which consists in the infusion of autologous or allogeneic lymphocytes upon ex vivo expansion and (in some instances) genetic engineering [2]. However, while autologous chimeric antigen receptor (CAR)-expressing T cells have rapidly become a mainstay for the treatment of various hematological malignancies [3], such an ACT variant is not yet licensed for the treatment of solid tumors, and no other forms of ACT has yet received regulatory approval for routine clinical use in cancer patients. Moreover, while > 80% of patients with hematological tumors receiving CAR-expressing T cells experience (often profound) objective responses, a sizeable proportion thereof ultimately relapse, often (but not always) due to the loss of the antigenic CAR target [4, 5]. Thus, there is ample room for improvement in the ACT field.

In this context, natural killer (NK) cells have attracted considerable attention as a potential form of ACT, largely due to their antigen-independent, robust cytolytic activity against cells that display specific surface features (**Box 1**) [6]. Abundant preclinical data indicate that NK cells not only participate in cancer immunosurveillance (at least in some tumors) [7–9], but also support therapeutic responses as elicited by a variety of treatments, including chemotherapy [10, 11], radiation therapy (RT) [12, 13], targeted anticancer agents [14, 15], and peptide-mediated oncolysis [16]. Moreover, signs of NK cell activation have been associated with improved clinical outcome in various oncological settings, including acute myeloid leukemia (AML) [17–19], gastrointestinal stromal tumors [20, 21] and breast cancer [22, 23].

Early clinical studies demonstrate that alloreactive NK cells can efficiently eliminate leukemic blasts in subjects with AML during haploidentical hematopoietic stem cell transplantation (HSCT), *de facto* extending patient survival [24–26]. The adoptive transfer of alloreactive NK cells has also shown encouraging clinical responses in patients with AML, refractory lymphoma, and advanced multiple myeloma outside of the HSCT setting [27–30]. More recently, CAR-expressing NK cells have been shown to mediate objective responses in eight of eleven patients with B cell malignancies, including four complete remissions [31]. However, while no less than 40 clinical trials are currently open to investigate the safety and efficacy of adoptively infused NK cells (often combined with other therapeutic modality) in patients with solid tumors (Table 1), signals of efficacy remain sporadic, as in the case of two distinct clinical trials reporting clinical benefits in neuroblastoma patients receiving allogeneic NK cells in combination with ganglioside D2 (GD2)-targeting antibodies [32, 33].

**Table 1:** Clinical trials currently testing NK cells as therapeutic agents in patients with solid tumors.*

Indication(s)	Phase	Status	Nº	Source	Other regimens	Notes	Ref.
Breast cancer	1	Recruiting	20	Allogenic	HER2 blockers IL2	In patients with HER2^+^breast cancer	NCT05385705
Breast cancer Gastric cancer	1	Recruiting	36	Allogenic	Single agent	Off-the-shelf product targeting HER2^+^ tumors	NCT04319757
BTC	2–3	Recruiting	128	Allogenic	PD-1 blocker	Multi-arm study	NCT05429697
CRC	1	Not yet recruiting	12	Allogenic	IL2 TGFB1 blocker	Single-arm study	NCT05400122
CRC	1	Recruiting	15	Allo﻿genic	Cetuximab	UCB-derived	NCT05040568
CRC	1	Recruiting	18	Auto﻿logous	Single agent	Expanded ex vivo by proprietary protocol	NCT05394714
CRC	1	Recruiting	38	N/A	Single agent	Engineered to express an NKG2D-like CAR	NCT05213195
CRC Sarcoma	1	Active, not recruiting	14	Allo﻿genic	IL15R agonist	Single-arm study	NCT02890758
Gastric cancer	N/A	Recruiting	18	Allo﻿genic	Single agent	UCB-derived	NCT04385641
GBM	1	Not yet recruiting	25	Allo﻿genic	Single agent	UBC-derived, engineered to resist TGFB1	NCT04991870
GBM	1	Recruiting	5	Auto﻿logous	Single agent	Intratumoral delivery	NCT05108012
GEJ tumors HNSCC	2	Recruiting	55	Allo﻿genic	IL15R agonist PD-1 blocker	Engineered to express a PD-L1-targeting CAR	NCT04847466
GIST	2	Not yet recruiting	1	Auto﻿logous	DCs PD-1 blocker	Single-arm study	NCT05461235
Glioblastoma	1	Recruiting	42	Allo﻿genic	PD-1 blocker	NK92 cell-based	NCT03383978
Glioma	1	Not yet recruiting	24	Auto﻿logous	Single agent	Single-arm study	NCT04254419
HCC	1–2	Recruiting	200	Allo﻿genic	SOC	Multi-arm study	NCT04162158
HCC	2	Recruiting	20	Auto﻿logous	5-fluorouracil Cisplatin	Single-arm study	NCT05040438
HCC	2	Recruiting	35	Allo﻿genic	Apatinib PD-1 blocker	UCB-derived	NCT05171309
HNSCC	1	Recruiting	12	Allo﻿genic	IL15R agonist CTLA4 blocker	Multi-arm study	NCT04290546
Neuroblastoma	1	Active, not recruiting	13	Auto﻿logous	GD2 blocker Lenalidomide	Single-arm study	NCT02573896
Neuroblastoma	1	Active, not recruiting	85	Allo﻿genic	GD2 blocker IL2	Single-arm study	NCT02650648
Neuroblastoma	1–2	Not yet recruiting	31	Auto﻿logous	GD2 blocker Irinotecan Temozolomide	Single-arm study	NCT04211675
Neuroblastoma	2	Active, not recruiting	153	Allo﻿genic	SOC	In the context of HSCT	NCT01857934
Neuroblastoma Sarcoma	2	Active, not recruiting	15	Allo﻿genic	Single agent	In the context of HSCT	NCT02100891
NSCLC	1–2	Recruiting	24	Allo﻿genic	Single agent	Dose-finding study	NCT04616209
NSCLC	1–2	Recruiting	24	Auto﻿logous	Carboplatin Cetuximab Gemcitabine	Multi-arm study	NCT04872634
NSCLC	1	Enrolling by invitation	5	Allo﻿genic	Single agent	NK92 cell-based	NCT03656705
NSCLC	1	Recruiting	20	Auto﻿logous	SOC	Including γδ T cells	NCT04990063
NSCLC	1	Recruiting	21	Allo﻿genic	PD-L1 blocker	UCB-derived, engineered to express IL15	NCT05334329
Prostate cancer	1	Recruiting	9	N/A	Single agent	Engineered to express a FOLH1-targeting CAR	NCT03692663
RTC	1–2	Recruiting	40	Auto﻿logous	Single agent	Engineered to express a CLDN6-targeting CAR	NCT05410717
Solid tumors	N/A	Recruiting	60	N/A	SOC	Multi-arm study	NCT04214730
Solid tumors	1	Active, not recruiting	12	Allo﻿genic	IL2 PD-L1 blocker	Dose-escalation plus expansion phase	NCT04551885
Solid tumors	1	Active, not recruiting	27	Auto﻿logous	PD-1 blocker PD-L1 blocker	Expanded ex vivo by proprietary protocol	NCT03941262
Solid tumors	1	Not yet recruiting	12	Auto﻿logous	Single agent	Dose-finding study	NCT04557306
Solid tumors	1	Recruiting	12	N/A	Oncolytic virus	Trained immunity NK cells	NCT05271279
Solid tumors	1	Recruiting	30	Auto﻿logous	IL15R agonist	Memory-cytokine enriched NK cells	NCT04898543
Solid tumors	1	Recruiting	37	Allo﻿genic	IL2 PD-1 blocker PD-L1 blocker	iPSC-derived	NCT03841110
Solid tumors	1	Recruiting	38	Allo﻿genic	Cyclophosphamide Etoposide	UCB-derived	NCT03420963
Solid tumors	1	Recruiting	40	N/A	Single agent	Engineered to express a TPBG-targeting CAR	NCT05194709
Solid tumors	1	Recruiting	40	Auto﻿logous	Single agent	Including NKT cells and CTLs	NCT05237206
Solid tumors	1	Recruiting	56	Allo﻿genic	Single agent	Engineered to express a TPBG-targeting CAR	NCT05137275
Solid tumors	1	Recruiting	189	Allo﻿genic	Cetuximab HER2 blocker IL2 PD-1 blocker PD-L1 blocker	Combinatorial regimens based on tumor type	NCT05069935
Solid tumors	1	Recruiting	322	Allo﻿genic	Cetuximab EGFR blocker HER2 blocker IL2 PD-1 blocker PD-L1 blocker	Combinatorial regimens based on tumor type	NCT05395052
Solid tumors	1–2	Recruiting	60	N/A	Decitabine	Post-remission	NCT05143125
Solid tumors	1–2	Recruiting	200	Autologous	Single agent	Activated ex vivo	NCT03634501

*Abbreviations.* BTC, biliary tract cancer; CAR, chimeric antigen receptor; CRC, colorectal carcinoma; CTL, cytotoxic T lymphocyte; DC, dendritic cell; GBM, glioblastoma; GEJ, gastroesophageal junction; GIST, gastrointestinal stromal tumor; HCC, hepatocellular carcinoma; HNSCC, head and neck squamous cell carcinoma; HSCT, hematopoietic stem cell transplantation; iPSC, inducible pluripotent stem cell; N/A, not available or not applicable; NK, natural killer; NKT, natural killer T; NSCLC, non-small cell lung carcinoma; RTC, reproductive tract cancer; SOC, standard-of-care; UCB, umbilical cord blood. *source http://www.clinicaltrials.gov; limited to studies with status “Not yet recruiting”, “Recruiting”, “Enrolling by invitation” and “Active, not recruiting”.

Clinical findings suggest that two parameters are critical for adoptively transferred NK cells to mediate therapeutically relevant effects in patients with solid tumors: (1) intratumoral accumulation [34], and (2) persistence in an activated state [28]. Here, we review metabolic and immunological features of the tumor microenvironment (TME) that prevent adoptively transferred NK cells from successfully infiltrating, persisting within and mediating effector functions against solid tumors as we critically discuss strategies to circumvent such barriers in support of superior therapeutic activity.

## Environmental obstacles for optimal NK cell anticancer activity

**Fig. 1:**
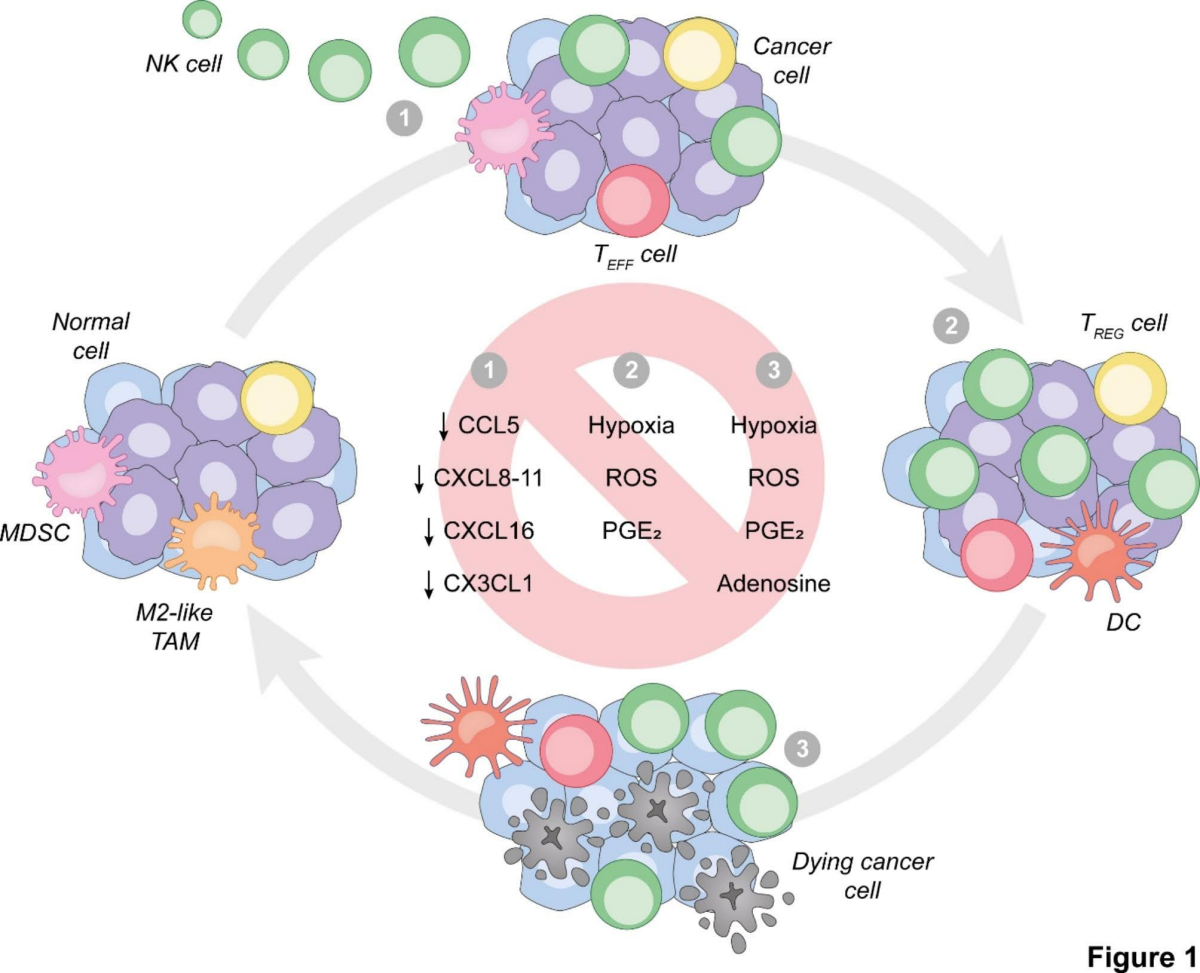
**Environmental obstacles against optimal NK cell activity in solid tumors.** For optimal anticancer effects, adoptively transferred natural killer (NK) cells must (1) access and abundantly infiltrate the tumor microenvironment, (2) persist and proliferate therein in the context of preserved NK cell-activating receptor expression and limited NK cell-inhibiting receptor expression, and (3) ultimately mediate potent secretory and cytotoxic functions. Moreover, malignant cells must retain expression of NK cell-activating ligands and sensitivity to the cytotoxic activity of NK cells. Defects in NK cell trafficking as well as environmental parameters including (but not limited to) hypoxia, reactive oxygen species (ROS), prostaglandin E_2_ (PGE_2_) secretion and extracellular adenosine abundance interfere with one or several of these *sine qua non*, ultimately limiting (and hence representing valid targets to improve) the therapeutic effects of adoptively transferred NK cells against solid tumors. CCL5, C-C motif chemokine ligand 5; CXCL, C-X-C motif chemokine ligand; CX3CL1, C-X3-C motif chemokine ligand 1; DC, dendritic cell; MDSC, myeloid-derived suppressor cell; TAM, tumor-associated macrophage; T_EFF_, effector T; T_REG_, regulatory T

A number of environmental obstacles prevent the accumulation of adoptively transferred NK cells into the TME of solid neoplasms, limit their persistence therein and/or inhibit their cytotoxic functions, including (but not limited to) impaired NK cell trafficking as well as metabolic TME features with potent immunosuppressive effects [35, 36] (Fig. 1).

***Impaired NK cell trafficking.*** NK cell trafficking and homing are regulated by various factors including integrins, selectins, and chemokine receptors [37]. Specifically, C-X-C motif chemokine receptor 3 (CXCR3) appears to play a major role in NK cell recruitment to solid tumors. In line with this notion, > 60% of NK cells infiltrating human breast cancer has been reported to express CXCR3 [38]. Moreover, the CXCR3 ligands C-X-C motif chemokine ligand 9 (CXCL9), CXCL10 and CXCL11, which are secreted in response to type I interferon (IFN) and interferon gamma (IFNG) signaling, have been mechanistically implicated in NK cell infiltration of experimental lung adenocarcinomas [39] lymphomas [40] and melanomas [41]. Thus, strategies aimed at enhancing CXCR3 expression by NK cells and/or secretion of CXCR3 ligands in the TME may result in increased NK cell recruitment to the TME of solid tumors. On the one hand, CXCR3 expression by NK cells has been shown to increase during ex vivo expansion in the presence of interleukin 2 (IL2) [41], but rather drop in the context of short-term IL2 stimulation [42]. Along with the considerable drawbacks of recombinant IL2 administration – including a non-negligible toxicity and the expansion of immunosuppressive CD3^+^CD4^+^CD25^+^FOXP3^+^ regulatory T (T_REG_) cells [43] – these observations delineate a benefit for ex vivo expansion prior to ACT over in vivo NK cell stimulation. On the other hand, a variety of strategies other than type I IFN or IFNG administration (which are also associated with considerable side effects and hence have been mostly abandoned) [44, 45] has been shown to promote the secretion of CXCR3 ligands in the TME. Specifically, the administration of dipeptidyl peptidase inhibitors has been reported to drive CXCL9 and CXCL10 secretion in experimental models of hepatocellular carcinoma (HCC) and pancreatic ductal adenocarcinoma (PDAC), culminating with CXCR3^+^ NK cell recruitment and (at least in some setting) synergy with immune checkpoint inhibitors (ICIs) [46, 47]. Similar results have been observed in models of colorectal carcinomas treated with curaxins (small molecules that interfere with DNA-histone interactions) [48], as well as in models of HPV-driven tumors responding to RT plus ATR serine/threonine kinase (ATR) inhibitors [49].

A number of genotoxic agents including RT are indeed able to drive a type I IFN response culminating with CXCL10 secretion via the accumulation of nuclear and mitochondrial DNA in the cytoplasm and consequent activation of cyclic GMP-AMP synthase (CGAS) [50–52]. Moreover, it has recently been reported both CXCL10 and C-C motif chemokine ligand 5 (CCL5) are abundantly secreted by glioblastoma (GBM) cells upon exposure to the lysosomal inhibitor chloroquine, resulting in accrued accumulation of adoptively transferred CAR-expressing NK cells in support of superior therapeutic activity [53]. Similar findings have been documented in preclinical models of melanoma [54] and non-small cell lung carcinoma (NSCLC) [55] receiving pharmacological inhibitors of autophagy or genetically engineered to become autophagy-deficient [56], culminating with accrued infiltration of endogenous NK cells and hence inhibited tumor growth [55]. Of note, the ability of autophagy inhibitors to promote NK cell chemotaxis downstream of CXCL10 and CCL5 hypersecretion has been linked (at least some settings) with superior CGAS signaling [57]. In line with this notion, pharmacological agonism of the CGAS signal transducer stimulator of interferon response cGAMP interactor 1 (STING1) has recently been shown to drive an abundant recruitment of CAR-expressing NK cells to mesothelioma organoids, culminating with potent tumor killing [58]. A similar improvement in tumor infiltration by natural or adoptively transferred NK cells has been documented in preclinical melanoma models secreting CCL5 downstream of viral infection [59], as well as in models of HCC receiving a CCL5-coding adenoviral vector [60].

Additional chemokine receptors that have been shown to promote NK cell chemotaxis and recruitment to the TME of solid tumors (at least in mice) include: (1) CXCR4, whose overexpression endowed adoptively transferred NK cells with superior homing capacities to GBM xenografts [61]; (2) C-C motif chemokine receptor 7 (CCR7), which upon acquisition via trogocytosis promotes lymph node homing [62, 63], and (3) chemokine (C-X3-C motif) receptor 1 (CX3CR1), which has been involved in superior NK cell responses driven in experimental melanomas and CRCs by the transgene-driven expression of its cognate ligand C-X3-C motif chemokine ligand 1 (CX3CL1) [64]. However, clinically viable strategies to drive the expression of CXCR4, CCR7 and CX3CR1 ligands in the TME remain to be identified. Moreover, at least in some oncological indications, high intratumoral levels of CX3CL1 have been associated with dismal prognosis [65], calling for at least some caution on strategies that would increase the intratumoral levels of this cytokine.

On the contrary, RT has been successfully employed to drive NK cell infiltration in experimental mammary tumors [66, 67] and PDACs [68], via a mechanism that involved CXCL16 and CXCL8 secretion, respectively. However, CXCL8 has been associated with immunoevasion, tumor progression and resistance to (immuno)therapy in a variety of oncological settings [69, 70]. Thus, promoting CXCL8 secretion by cancer cells might not only favor the recruitment of NK cells but also promote tumor infiltration by immunosuppressive cells that may offset therapeutic efficacy. Conversely, many tumors express high levels of CXCL8 at baseline [71], pointing to the transgene-driven overexpression of CXCR1 and CXCR2 (the main CXCL8 receptors) as a feasible approach to action the CXCL8 axis in support of NK cell recruitment to the TME of solid tumors. Preclinical data in support of this possibility have already been obtained in models of renal cell carcinoma (RCC) [72] and ovarian cancer [73].

Taken together, these data suggest that NK cell trafficking to solid malignancies can be ameliorated by various strategies that prime the TME to secrete increased amounts of NK cell-targeting chemokines or by genetically engineering NK cells to overexpress relevant chemokine receptors.

***Hypoxia.*** The TME of solid tumors is frequently hypoxic owing to defects in vasculature coupled to increased local oxygen demand [74, 75], which is toxic for tumor-infiltrating lymphocytes, particularly NK cells [76, 77]. Indeed, hypoxia has a variety of detrimental effects on NK cells, including a transcriptional rewiring accompanied by the downregulation of multiple NK cell-activating receptors and effector molecules, but less so NK cell-inhibitory receptors, cytokine receptors or the receptors that mediate antibody-dependent cellular cytotoxicity (ADCC) (**Box 1**) [78–81]. At least in part, this originates from the ability of hypoxia to potently inhibit mitogen-activated protein kinase 1 (MAPK1, best known as ERK) signaling in NK cells, resulting in reduced transcription of signal transducer and activator of transcription 3 (STAT3) target genes [79] coupled with limited sensitivity to NK cell-activating stimuli including phorbol 12-myristate 13-acetate (PMA) plus ionomycin, as well as IL-15 and IL-18 [81]. Moreover, hypoxia promotes the expression of various surface proteins that impair NK cell functions via killer immunoglobulin-like receptors (KIRs) and other co-inhibitor receptors (**Box 1**), such as major histocompatibility complex, class I, G (HLA-G) and CD274 (best known as PD-L1) [82, 83].

Finally, at least in some setting, hypoxia causes mitochondrial fragmentation in NK cells via a pathway involving constitutive mechanistic target of rapamycin (MTOR) signaling and consequent activation of dynamin 1 like (DNM1L, best known as DRP1), resulting in a shift from oxidative phosphorylation to glycolysis and compromised tumor control [84]. On the contrary, glycolysis appears to be critical for NK cells to properly control viral infections [85]. Whether this apparent discrepancy reflects the particularly disadvantageous conditions of the solid TME remains to be elucidated. Interestingly, despite being poorly cytotoxic, NK cells lacking hypoxia inducible factor 1 subunit alpha (HIF1A), a master transcription factor for hypoxia adaptation [86], have been shown to mediate anticancer effects in vivo by antagonizing vascular endothelial growth factor A (VEGFA)-driven vascularization [87]. That said, the molecular alterations driven by hypoxia via HIF1A in NK cells remain to be elucidated.

Importantly, hypoxia also reduces cancer cell sensitivity to NK cell killing, via numerous mechanisms. For example, hypoxic breast cancer cells exhibit an increased autophagic flux resulting in superior granzyme B (GZMB) (**Box 1**) degradation [88]. A similar mechanism has been shown to originate by pseudohypoxia as driven by *VHL* mutations in RCC cells, resulting in accrued autophagic flux via the endothelial PAS domain protein 1 (EPAS1)-dependent upregulation of inositol 1,4,5-trisphosphate receptor type 1 (ITPR1) [89]. Moreover, hypoxia has been shown to downregulate the expression of NK cell-activating ligands (**Box 1**) on the surface on NSCLC and prostate cancer cells, at least in some settings via a HIF1A-dependent mechanism, culminating in limited NK cell activation [90, 91]. Tumor-derived microvesicles (TD-MVs) produced under hypoxic conditions are enriched in transforming growth factor beta 1 (TGFB1) and miR-23a, and hence suppress NK cell activity upon uptake by downregulating NK cell-activating receptors (**Box 1**) as well as the effector molecule lysosomal-associated membrane protein 1 (LAMP1, best known as CD107a) [92].

Finally, hypoxic tumor regions are enriched in a variety of immunosuppressive cells that interfere with NK cell functions (see below), including (but not limited to): T_REG_ cells [93–95], M2-like tumor-associated macrophages (TAMs) [96, 97] and myeloid-derived suppressor cells (MDSCs) [98–100].

Reversing hypoxia stands out as a potential strategy to restore NK cell functions in the TME of solid tumors. Some studies have suggested that physical exercise may improve oxygenation in the TME and hence reverse, at least partially, hypoxia [101]. However, the ability of exercise training to restore NK cell functions in cancer patients remain to be formally demonstrated [102]. Conversely, myo-inositol-trispyrophosphate (ITPP), which increases oxygen liberation by hemoglobin, has been shown to increase NK cell abundance while decreasing T_REG_ cell numbers in the TME of experimental melanomas [103]. Similar results have been obtained with human breast cancer spheroids treated with manganese dioxide nanoparticles encapsulated into polylactic-co-glycolic acid, which degrade tumor-derived hydrogen peroxide into molecular oxygen [104]. Of note, in this latter setting, HIF1A downregulation was accompanied by other favorable alterations of the TME, including reactive oxygen species (ROS), lactate and adenosine reductions [104].

An alternative approach to circumvent the detrimental effects of hypoxia on NK cells consists in rendering the latter more tolerant to low oxygen levels. At least in some setting, IL2 priming has been shown to prevent the hypoxia driven downregulation of killer cell lectin like receptor K1 (KLRK1, best known as NKG2D) [80]. Along similar lines, NK cells engineered to overexpress CD16 (**Box 1**) and IL2 preserve their ability to mediate ADCC and antibody-independent cytotoxicity in hypoxic microenvironments [105]. Finally, pharmacological inhibition of the ERK phosphatase protein tyrosine phosphatase non-receptor type 6 (PTPN6, best known as SHP-1) has been shown to efficiently prevent hypoxia-driven ERK-STAT3 silencing and consequent NK cell dysfunction [79]. The latter approach, however, may result in the compensatory expression of PD-L1, which in models of prostate cancer has been shown to support (rather than prevent) NK cell dysfunction while providing a therapeutic target for ICIs [91]. Finally, autophagy inhibition has been shown to restore the sensitivity of hypoxic cancer cells to NK cell-dependent cytotoxicity [88]. However, clinically viable pharmacological autophagy inhibitors remain elusive [106]. Moreover, NK cells are critically dependent on autophagy for their development and activity [107], in thus far resembling most other immune effector cells [108], overall casting doubts on non-targeted autophagy inhibition as a viable strategy to restore cancer cell sensitivity to lysis by NK cells.

In summary, increasing oxygen availability in the TME or rendering NK cells resistant to hypoxia stand out as the most promising strategies to circumvent the detrimental effects of poor oxygen availability on NK cell functions.

***Reactive oxygen species.*** ROS are abundant in the TME of solid tumors and promote disease progression via a variety of mechanisms including immunoevasion [109–111]. Indeed, while malignant cells as well as immunosuppressive M2-like TAMs are generally endowed with efficient mechanisms for ROS detoxification [112, 113], non-transformed cells as well as immune effector cells including NK cells are particularly sensitive to the genotoxic and cytotoxic effects of ROS [114].

Besides overt cytotoxicity, which only emerges in the presence of high ROS levels, one of the mechanisms through which ROS impair the anticancer activity of NK cells involve alterations in NK cell membrane properties. Specifically, ROS promote the accumulation of anionic charges on the surface of NK cells, limiting their ability to adhere to similarly charged target cancer cells, a defect that can be prevented by antioxidant molecules including superoxide dismutase (SOD) mimetics and catalase (CAT) [115]. Moreover, ROS species produced by cytochrome b-245, beta polypeptide (CYBB, best known as NOX2) have been mechanistically implicated in the ability of experimental melanomas to form metastasis via a mechanism that (1) is manifest only in immunocompetent (but not IFGN deficient) hosts, and (2) involves NK cell dysfunction [116]. At least in part, this may reflect the ability of ROS to downregulate CD16 on the surface of (and hence impair ADCC by) NK cells [117]. Finally, ROS have also been shown to promote the accumulation of M2-like macrophages, which also limit NK cell activation [118].

Increasing the tolerance of NK cells to ROS stands out as a promising approach to improve their anticancer effects in the TME of solid tumors beyond ROS scavenging [115] and inhibition of ROS-producing systems [116]. For example, IL15 – which is a potent NK cell activator [119] – has been reported to upregulate thioredoxin (TXN) in NK cells via an MTOR-dependent mechanism that increases the availability of reducing thiols on the cell surface, culminating with preserved cytotoxic functions despite environmental oxidative stress [120]. Similar results have been obtained with an activator of NFE2 like bZIP transcription factor 2 (NFE2L2, a master regulator of antioxidant responses best known as NRF2) [121] in NK cells from healthy donors [122], as well as by engineering CAR-expressing T cells specific for erb-b2 receptor tyrosine kinase 2 (ERBB2, best known as HER2) to overexpress CAT, which resulted not only in superior cytotoxicity against HER2-expressing mammary tumors, but also in preserved bystander cytotoxicity by otherwise ROS-sensitive NK cells [123].

These observations exemplify strategies that might be employed to limit the detrimental effects of ROS on NK cells adoptively transferred for the treatment of solid tumors.

***Prostaglandin E***_***2***_. A variety of tumors emerge and progress in the context of a chronic, indolent inflammatory response that ultimately promote immunoevasion, which is commonly known as tumor-promoting inflammation (TPI) [124]. Prostaglandin-endoperoxide synthase 1 (PTGS1, best known as COX1) and PTGS2 (best known as COX2) are key contributors to TPI as they secrete the mitogenic and immunosuppressive factor eicosanoid prostaglandin (prostaglandin E_2_) [125–127].

While mouse splenic NK cells express all four main PGE_2_ receptors, it appears that prostaglandin E receptor 2 (PTGER2, best known as EP_2_) and even more so PTGER4 (EP_4_) are the main transducers of PGE_2_-elicited immunosuppression [128, 129]. Indeed, selective EP_4_ agonists have been shown to efficiently inhibit both IFNG production and chemotactic responses to serum chemokines by mouse NK cells, while EP_2_ agonists only had partial suppressive activity [128]. Moreover, pharmacological inhibition of EP_4_ reportedly protects NK cells from the immunosuppressive effects of PGE_2_ in preclinical models of mammary carcinomas, resulting in preserved effector functions and antimetastatic activity [129]. Similar findings have been obtained in models of CRC [130].

Mechanistically, PGE_2_ produced by HCC cells has been shown to cooperate with products of the immunosuppressive enzyme indoleamine 2,3-dioxygenase 1 (IDO1) at inducing the downregulation of NKG2D and other NK cell-activating receptors (**Box 1**) in tumor-infiltrating human NK cells, resulting in profound dysfunction [131]. Along similar lines, PGE_2_ secretion by BRAF-mutant melanoma cells considerably reduces the viability of mouse NK cells recruited to the TME, as well as their capacity to secrete CCL5 and X-C motif chemokine ligand 1 (XCL1) [132], which is critical for the recruitment of cross-presenting dendritic cells (DCs) and hence for the initiation of tumor-targeting adaptive immune responses that can be therapeutically actioned with ICIs [133]. Finally, tumor-derived PGE_2_ mediates indirect immunosuppressive effects by an EP_2_- and EP_4_-elicited, ERK-dependent mechanism whereby tumor-infiltrating MDSCs are activated to produce increase levels of TGFB1, culminating with NK cell dysfunction in both human and mouse experimental systems [134, 135].

EP_4_ inhibitors stand out as a potential strategy to prevent NK cell dysfunction driven by PGE_2_ [129]. Alongside, IL15 has been shown to endow NK cells with resistance to PGE_2_ and preserved anticancer activity, both in vitro and in vivo, as a function of sustained MTOR signaling coupled with phosphodiesterase 4 A (PDE4A) expression and CD25/CD54 co-expression [136]. Whether any of these proteins can be directly targeted to limit NK cell suppression by PGE_2_, however, remains to be elucidated.

***Adenosine.*** Adenosine is a ubiquitous metabolite generated as the terminal product of ATP degradation [137]. Adenosine accumulates in the extracellular milieu in the context of immunogenic cell stress and death as imposed by the (natural or treatment-driven) adverse microenvironmental conditions of the TME [138, 139], which is coupled to abundant ATP release, thanks to the sequential activity of two nucleotidases: (1) ectonucleoside triphosphate diphosphohydrolase 1 (ENTPD1, best known as CD39), which degrades ATP into ADP and AMP, and (2) 5’-nucleotidase ecto (NT5E, best known as CD73), which degrades AMP into adenosine [140, 141]. CD39 and CD73 are overexpressed by malignant cells as well both myeloid [142, 143] and lymphoid [144, 145] components of the TME in a variety of solid tumors, resulting in constitutively high extracellular adenosine levels [146, 147].

Adenosine mediates broad immunosuppressive effects upon binding to adenosine A2a receptor (ADORA2A, also known as A_2_AR) or ADORA2B (also known as A_2_BR) on the surface of immune effector cells [148]. Specifically, adenosine has been shown to suppress both cytokine release [149] and cytotoxic functions [150, 151] in activated NK cells via an A_2_AR-initiated, cyclic AMP (cAMP)-dependent signaling cascade resulting in protein kinase A (PKA) engagement [149–151]. At least in some setting, such a dose-dependent inhibitory effect [152] manifest with decreased expression of CD56 [153], Fas ligand (FASL) and perforin 1 (PRF1) (**Box 1**) [150], as well as with limited IFNG and TNF secretion [149].

Of note, tumor-infiltrating lymphocytes including NK cells generally express higher levels of CD39, CD73 and/or CD38 (yet another adenosine-producing enzyme) [154, 155] as compared to their circulating counterparts [153, 156, 157], which (at least in some settings) results in the acquisition of immunosuppressive properties. Specifically, CD73^+^ NK cells infiltrating breast carcinomas and sarcomas have been shown to express a number of immunosuppressive molecules including (but not limited to) PD-L1, PD-1, lymphocyte activating 3 (LAG3), hepatitis A virus cellular receptor 2 (HAVCR2, best known as TIM-3) and T cell immunoreceptor with Ig and ITIM domains (best known as TIGIT) [158], correlating with abundant secretion of immunosuppressive cytokines such as IL10 and TGFB1 [156]. Along similar lines, CD56^bright^CD16^−^ NK cells (but not their CD56^dim^ counterparts) express not only CD38 but also ectonucleotide pyrophosphatase/phosphodiesterase 1 (ENPP1, which is required for adenosine synthesis downstream of CD38), and hence can exert potent immunosuppressive effects on other immune effectors – including CD4^+^ helper T lymphocytes – as a consequence of abundant adenosine production [159]. At least in part, the detrimental effects of adenosine on NK cells also involve the activation of tumor-resident immunosuppressive cells including M2-like TAMs, MDSCs and T_REG_ cells [160, 161].

Corroborating the potent immunosuppressive activity of adenosine on NK cells, both pharmacological and genetic strategies aimed at interrupting A_2_AR and/or A_2_BR activation have been shown to prevent adenosine-driven NK cell dysfunction in a variety of experimental settings [149–152]. Along similar lines, *Entpd1* or *Nte5* deletion as well as pharmacological or antibody-mediated inhibition of CD39 and/or CD73 have been consistently associated with restored NK cell activity and improved tumor control in numerous preclinical models of malignancy, including melanoma [162], sarcoma [163], glioblastoma [53], as well as prostate [163], breast [164], and colorectal cancer [165, 166]. In the context of ACT, CD73 blockage has been shown to promote the recruitment of CAR-expressing NK cells engineered to display increased levels of NKG2D to NSCLC xenografts [167]. Moreover, IL15 appears to be superior to IL2 to render NK cells expanded ex vivo resistant to adenosine [168]. That said, while cytokine administration in the context of ACT promotes NK cell expansion and persistence, this approach is associated with various problems including (but not limited to) non-negligible toxicity and the expansion of (adenosine-producing) T_REG_ cells [43, 169].

Taken together, these observations suggest that inhibiting adenosine receptors and/or adenosine-producing enzymes stands out as a promising strategy to improve NK cell activation in the TME of solid tumors.

## Immunological and stromal barriers against NK cell activity

**Fig. 2:**
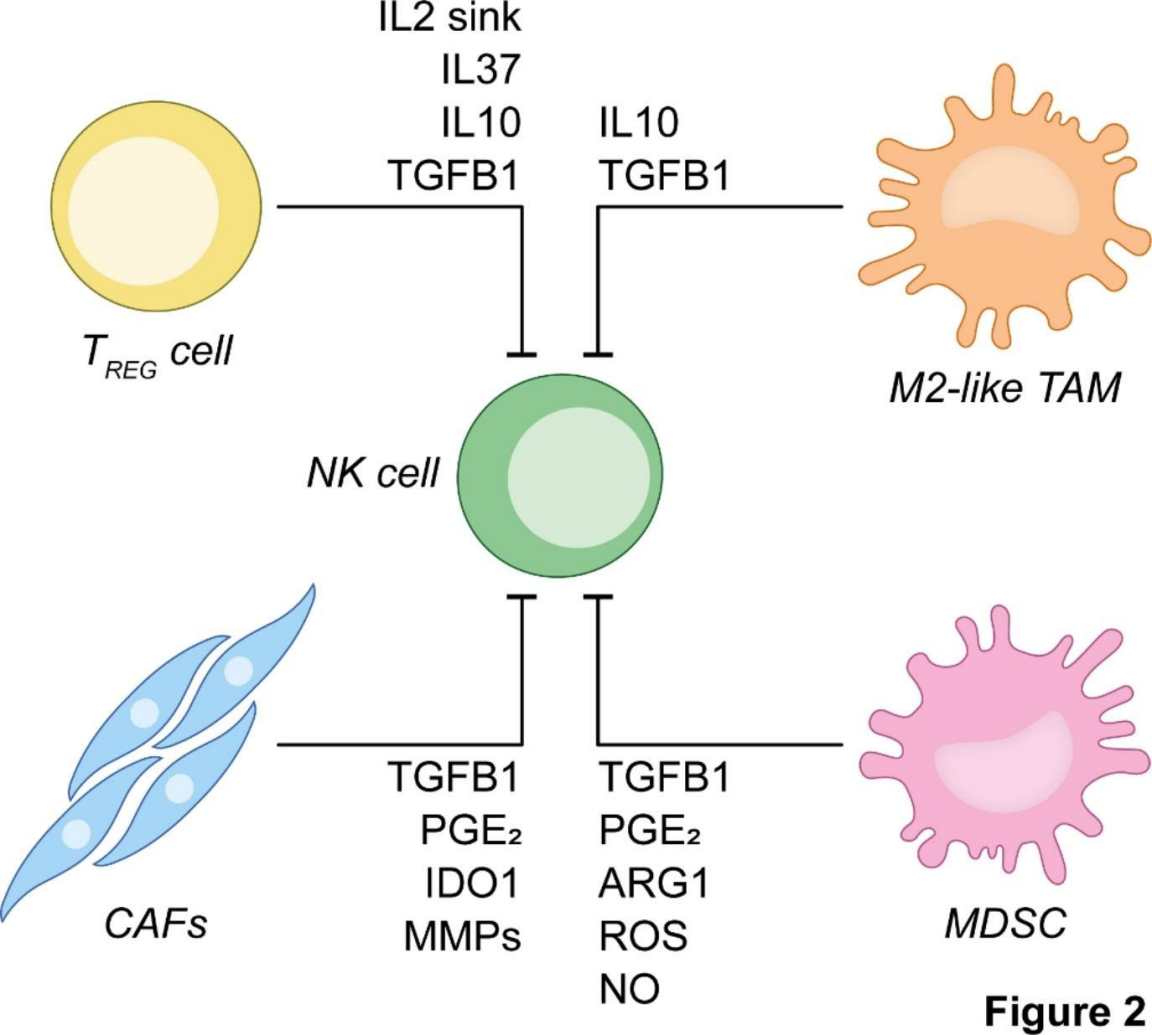
**Immunological and stromal barriers against optimal NK cell activity in solid tumors.** Tumor-infiltrating natural killer (NK) cells engage in contact-dependent and independent interactions with a variety of cells that ultimately inhibit their anticancer activity. Such cells include not only regulatory T (T_REG_) cells, M2-like tumor-associated macrophages (TAMs) and myeloid-derived suppressor cells (MDSCs), but also cancer-associated fibroblasts (CAFs). ARG1, arginase 1; IDO1, indoleamine 2,3-dioxygenase 1; IL, interleukin; MMP, metalloprotease; NO, nitric oxide; PGE_2_, prostaglandin E_2;_ ROS, reactive oxygen species; TGFB1, transforming growth factor beta 1

A number of immune and stromal cellular compartments of the TME potently inhibit the effector functions of NK cells. Thus, targeting these cell populations – which include T_REG_ cells, TAMs, MDSCs and cancer-associated fibroblasts (CAFs) – represents a promising approach to endow tumor-infiltrating NK cells with superior effector functions (Fig. 2).

***Regulatory T cells.*** Tumor-infiltrating T_REG_ cells mediate potent immunosuppressive effects via direct, contact-dependent pathways, as well as through direct and indirect humoral mechanisms [170]. For instance, T_REG_ cells have been shown to kill effector T (T_EFF_) cells upon GZMA and GZMB secretion, compete with T_EFF_ cells for IL2 availability (because T_REG_ cells express high levels of the high affinity IL2 receptor CD25) and secrete immunosuppressive cytokines including IL10 and TGFB1 [170]. In line with this notion, high intratumoral levels of T_REG_ cells have been associated with poor disease outcome in numerous cohorts of patients with cancer [171]. Moreover, a number of pharmacological and genetic strategies for T_REG_ cell depletion, including CD25-targeting antibodies [172, 173] as well as the expression of the diphtheria toxin (DT) receptor under the control of the *Foxp3* promote coupled to DT administration [174, 175], have been shown to improve the efficacy of various anticancer regimens in mice, generally in the absence of overt autoimmune reactions.

Both human and mouse canonical NK cells are highly sensitive to T_REG_ cell-mediated immunosuppression, generally resulting in decreased expression of NK cell-activating receptors such as NKG2D (**Box 1**), upregulation of co-inhibitory receptors such as PD-1 and interleukin 1 receptor accessory protein like 1 (IL1RAPL1, best known as IL1R8), coupled to limited proliferative and cytotoxic responses upon activation [176–178]. This has been shown to translate into limited control of primary tumor growth and metastatic dissemination in models of NSCLC and PDAC, through a mechanism that relies on STAT3 signaling in T_REG_ cells and TGFB1 secretion [176, 179]. At least in some settings, NK cells can also be made resistant to the immunosuppressive effects of T_REG_ cells upon exposure to IL2, IL7 or IL12 as well as neutralization of the IL1R8 ligand IL37 [176, 178, 180]. Moreover, adaptive NK cells that develop in the context of cytomegalovirus infection appear to be naturally insensitivity to T_REG_ cell-mediated immunosuppression [178]. At least in part, this phenomenon results from stable epigenetic modifications resulting in the expression of multiple NK cell-activating receptors and (paradoxically) TIM-3 coupled to the loss of NK cell-inhibiting receptors (**Box 1**), ultimately endowing adaptive NK cells with potent effector functions despite their terminally differentiated state [181–183]. Finally, a NK cell line engineered to express a chimeric receptor encompassing the extracellular domain of transforming growth factor beta receptor 2 (TGFBR2) fused to the intracellular domain of NKG2D has recently been shown to mediate superior therapeutic efficacy in preclinical HCC models, reflecting not only improved cytotoxic responses (which could further be ameliorated by TGFB1, at least in vitro), but also (1) enhanced recruitment to the solid TME, and (2) suppressed T_REG_ cell differentiation [184].

In summary, although therapeutically inhibiting or depleting T_REG_ cells in patients remain challenging [185], these immunosuppressive components of the TME stand out as promising targets to improve the efficacy of adoptively transferred NK cells against solid tumors.

***Tumor-associated macrophages.*** Human solid tumors are abundantly infiltrated by TAMs, often (but not always) driven by the CCL2-dependent recruitment of circulating monocytes [97, 186]. TAMs are a very plastic component of the TME that can adapt a spectrum of phenotypic and functional features ranging from a predominantly pro-inflammatory (M1-like) state (which is promoted by IFNG and TNF) to a prominently anti-inflammatory (M2-like) state (which is promoted by IL4, TGFB1 and PGE_2_) [97]. M2-like macrophages not only mediate robust immunosuppressive effects via contact-dependent (e.g., PD-L1 expression) and independent (e.g., TGFB1 and IL10 secretion; arginine depletion) mechanisms, but also promote neo-angiogenesis and metastatic tumor dissemination, *de facto* supporting disease progression and resistance to treatment in variety of oncological settings [187]. Accordingly, abundant tumor infiltration by TAMs as well as elevated circulating levels of TAM-relevant cytokines including CCL2, CCL8 and colony stimulating factor 1 (CSF1) have been linked with poor disease outcome in multiple cohorts of cancer patients [171, 187, 188]. Moreover, an abundant preclinical literature demonstrates that depleting M2-like TAMs or inhibiting their immunosuppressive functions mediates robust anticancer effects in mice, either as a standalone therapeutic strategy or combined with other antineoplastic regimens [187, 189].

M2-like TAMs isolated from spontaneous mouse mammary carcinomas as well as differentiated ex vivo from the peritoneum or bone marrow of healthy mice have been shown to potently inhibit NK cell cytotoxicity coupled to the acquisition of an exhausted CD27^low^CD11b^high^ phenotype via a TGFB1-dependent mechanism [190]. At least in preclinical CRC models, such an immunosuppressive pathway is initiated by the CAF-driven, CXCL8-dependent polarization of TAMs towards an M2-like phenotype [191]. M2-like TAMs collected from the ascites of patients with ovarian cancer and exposed to Toll-like receptor (TLR) ligands appear to undergo repolarization towards an M1-like state, resulting in IL12 secretion and acquisition of cytolytic functions by co-cultured NK cells [192, 193]. Finally, monoclonal antibodies targeting scavenger receptors on M2-like TAMs have been shown to limit their immunosuppressive effects and efficiently derepress the cytolytic functions of NK cells in human and mouse models of melanoma [194].

Despite the scarcity of studies directly investigating the interactions between TAMs and NK cells, these observations suggest that M2-like TAMs may also offer targets to improve the activity of adoptively transferred NK cells against solid tumors. That said, no agent conceived to deplete M2-like TAMs or repolarize them into their M1-like counterparts is available for clinical use yet.

***Myeloid-derived suppressor cells.*** MDSCs are a heterogenous population of immature bone marrow-derived myeloid cells with prominent immunosuppressive effects [195]. Human MDSCs are generally subdivided into CD11b^+^CD14^+^CD33^+^HLA-DR^low/neg^ monocytic (M)-MDSCs or CD11b^+^CD15^+^HLA-DR^low^CD66b^+^ granulocytic (G)- or polymorphonuclear (PMN)-MDSCs [196]. MDSCs expand peripherally in both cancer patients and tumor-bearing mice, at least in part driven by the systemic effects of cancer cell-derived cytokines that influence hematopoiesis in the bone marrow, including IL6 and CSF2 [197]. Moreover, MDSCs can accumulate in the TME of solid tumors upon recruitment via chemokines including (but not limited to) CCL2 [99, 195].

In tumor-bearing mice, the frequency of CD11b^+^Gr1^+^ MDSCs inversely correlates with the expression of NK cell-activating receptors including NKG2D and natural cytotoxicity triggering receptor 3 (NCR3, best known as NKp30) on the NK cell surface, as well as with IFNG and PRF1 production [198, 199]. At least in preclinical models, the ability of MDSCs to suppress NK cell functions requires physical contact, which is facilitated by membrane-bound TGFB1 [200]. In line with this notion, depleting MDSCs (but not T_REG_ cells) has been shown to restore NK cell-dependent tumor control in an orthotopic model of HCC [199]. Additional mechanisms through which MDSCs inhibit NK cells (as well as T_EFF_ cells) include the production of ROS and reactive nitrogen species (see above), as well as the depletion of essential amino acids such as arginine, reflecting the elevated expression of arginase 1 (ARG1) [195].

Several strategies to inhibit MDSCs in support of superior NK cell activity have been explored. For example, inhibiting MDSC trafficking by targeting CXCR1 and CXCR2 has been shown to efficiently limit MDSC recruitment in preclinical models of head and neck cancer (HNC), resulting in superior efficacy from adoptively transferred NK cells [198]. Along similar lines, inhibition of nitric oxide (NO) production by MDSCs with a nitric oxide synthase 2 (NOS2) inhibitor reportedly restores NK-cell mediated ADCC in preclinical models of breast cancer, resulting in superior tumor control in vivo [117]. Similar benefits have been documented with pharmacological inhibitors of ARG1 in preclinical models of CRC treated with adoptively transferred NK cells [201]. Additional strategies that efficiently inhibit MDSCs resulting in derepressed NK cell activity include specific chemotherapeutic agents (especially, doxorubicin, gemcitabine and low-dose cyclophosphamide) [202–206], approaches to prevent PGE_2_ secretion by MDSCs [134], as well as all-*trans* retinoic acid (ATRA), which is known to promote MDSC differentiation and hence limit their immunosuppressive activity [207]. Interestingly, ATRA can also promote the expression of NK cell-activating ligands including MHC class I polypeptide-related sequence A (MICA) and MICB on malignant cell, *de facto* rendering them more susceptible to NK cells [208].

Taken together, these observations delineate multiple, clinically viable strategies for targeting MDSCs to improve the activity of NK cell-based ACT in patients with solid tumors.

***Cancer-associated fibroblasts.*** Solid tumors are abundantly infiltrated by a highly plastic and functionally heterogeneous population of CAFs, which can originate from a variety of tissue-resident cells as well as from circulating precursors [209–211]. Several factors have been shown to promote the accumulation of CAFs in the TME of solid tumors including not only cytokines such as TGFB1 [212], CXCL12 [213] and platelet derived growth factor (PDGF) [214], but also oxidative stress coupled to mitochondrial dysfunction [215]. In multiple settings, CAFs have been shown to express fibroblast activation protein alpha (FAP), actin alpha 2, smooth muscle (ACTA2, best known as αSMA), S100 calcium binding protein A4 (S100A4, best known as FSP1), vimentin (VIM), and both subunits of the heterodimeric PDGF receptor [209].

Early studies demonstrated that CAFs promote tumor progression and resistance to treatment by a number of mechanisms, such as (1) favoring neoangiogenesis, (2) generating a dense stromal reactions that hinder tumor infiltration by drugs and immune effector cells, as well as (3) directly inhibiting the activity of the latter, notably NK cells [209, 216]. Specifically, CAFs produce abundant TGFB1 [217], which is known to potently suppress NK cell functions [218]. Moreover, fibroblasts exposed to melanoma, CRC or HCC cells secrete PGE_2_ and express IDO1, hence acquiring the ability to promote NKG2D and NKp30 downregulation on NK cells, coupled with suppressed cytotoxic activity [131, 219, 220]. Along similar lines, CAFs isolated from patients with endometrial cancer have been shown to potently suppress NK cell activity along with the downregulation of PVR cell adhesion molecule (PVR), yet another NK cell-activating receptor [221]. Finally, melanoma-associated CAFs have been reported to secrete metalloproteases that efficiently shed MICA and MICB from the surface of malignant cells, thus rendering them less prone to activate NK cells upon contact [222, 223].

Targeting FAP-expressing CAFs or TGFB1 signaling has been shown to mediate potent anticancer effects in a variety of preclinical tumor models [212, 224–226]. At least in models of PDAC, the beneficial effects of CAF-targeting strategies have been shown to originate from NK cell (rather than T_EFF_ cell) reactivation [227]. Moreover, NK cells can contribute to CAF inhibition when ADCC-competent CAF-targeting monoclonal antibodies are employed, as demonstrated in models of CRC where CAFs express abundant epidermal growth factor receptor (EGFR) levels [228].

In summary, while CAFs may represent valid targets to improve the activity of NK cells in the solid TME, the lack of specific and reliable CAF markers poses a non-negligible obstacle to this approach.

## Concluding remarks and future perspectives

While NK cells are attracting considerable interest as potential anticancer therapeutics and encouraging data have been documented in patients with hematological malignancies, the use of NK cells for the treatment of solid tumors remains hindered by a number of obstacles, as amply discussed herein [229–231]. Indeed, while numerous strategies aimed at improving the recruitment of NK cells to the TME as well as their persistence and activation have been proven effective in preclinical tumor models (see above), some of these approaches are complex to translate into clinically viable procedures (e.g., systemic infusion of NK cell-activating cytokines). On the contrary, priming the TME with (ideally FDA-approved) agents, such as low-dose chemotherapy to deplete T_REG_ cells and MDSCs [232] or RT to jumpstart anticancer immunity [233], stands out as a powerful and clinically valid approach to provide adoptively transferred NK cells with a relatively more permissive microenvironment. Alongside, incorporating specific agents (e.g., difference cytokines as well as modifications or combinations thereor) in ex vivo expansion protocols may offer a safe and yet powerful approach to create NK cell populations with increased resistance to the adverse metabolic and immunological conditions of the TME, such as cytokine-induced memory-like NK cells, in support of superior treatment efficacy. Finally, NK cells resemble T_EFF_ cells in expressing a number of co-inhibitory receptors that may targeted for therapeutic purposes, including (but not limited to) PD-1 and killer cell lectin like receptor C1 (KLRC1, best known as NKG2A). While only PD-1/PD-L1- and cytotoxic T lymphocyte-associated protein 4 (CTLA4)-targeting agents are approved for use in patients with cancer nowadays [234], several other ICIs are currently in clinical development including NKG2A-, LAG3- and TIM-3 blockers [234]. These agents may also offer a safe and convenient approach to boost the activity of adoptively transferred NK cells against solid tumors [235, 236].

In conclusion, while additional work is required, we surmise that combinatorial strategies aimed at enabling robust tumor infiltration and protecting NK cells from the metabolic and immunological conditions of the TME are key to unlock the therapeutic potential of adoptively transferred NK cells for human solid tumors.

## Box 1 - principles of NK cell biology

Human natural killer (NK) cells are CD45^+^CD3CD56^+^ cells that originate in the bone marrow and mature in peripheral lymphoid and non-lymphoid organs [237]. The ability of NK cells to proliferate, secrete cytokines and mediate cytotoxic effects is not regulated by an antigen-specific receptor as in the case of T and B lymphocytes, but is rather controlled by a balance between activating and inhibitory signals elicited by antigen-independent interactions with other cells [238]. Such signals are dispatched to NK cells by a variety of surface receptors including: (1) killer immunoglobulin-like receptors (KIRs), which generally deliver inhibitory cues via immunoreceptor tyrosine-based inhibitory motifs (ITIMs); (2) c-type lectin receptors, such as the immunosuppressive receptor killer cell lectin like receptor C1 (KLRC1, best known as NKG2A) and the immunostimulatory receptor KLRK1 (best known as NKG2D); (3) leukocyte immunoglobulin-like receptors (LIRs), such as the immunosuppressive molecule leukocyte immunoglobulin like receptor B1 (LILRB1), and (4) natural cytotoxicity receptors, such as natural cytotoxicity triggering receptor 1 (NCR1, best known as NKp46) and NCR2 (best known as NKp44), which deliver activating stimuli via immunoreceptor tyrosine-based activation motifs (ITAMs) [239].

NK cells also express (1) various cytokine receptors, notably the receptors for interleukin 12 (IL12), IL15 and IL21, which deliver mitogenic signals [240]; (2) at least some of the co-inhibitory receptors that suppress effector T cell activation, such as programmed cell death 1 (PDCD1, best known as PD-1) and T cell immunoreceptor with Ig and ITIM domains (best known as TIGIT) [239]; as well as (3) high affinity receptors for immunoglobulins, notably Fc gamma receptor IIIa (FCGR3A) and FCGR3B (the heterodimeric CD16 receptor), which underlie their ability to mediate antibody-dependent cellular cytotoxicity (ADCC) against opsonized cells [241, 242]. Besides ADCC, NK cells can harness at least three additional mechanisms to mediate cytotoxic effects: (1) the exocytic release of granules containing perforin 1 (PRF1) and various members of the granzyme protease family; (2) the secretion of interferon gamma (IFNG) and tumor necrosis factor (TNF); and (3) the engagement of death receptors including Fas cell surface death receptor (FAS) and TNF receptor superfamily member 10b (TNFRSF10B, best known as TRAIL-R2) on the surface of target cells [243]. Moreover, at least in some settings, NK cells secrete chemotactic factors for dendritic cells (DCs), such as X-C motif chemokine ligand 1 (XCL1) and C-C motif chemokine ligand 5 (CCL5), ultimately promoting the initiation of antigen-specific immune responses downstream of T-cell cross-priming [132]. Importantly, all these functions exhibit considerable degree of heterogeneity across diverse NK cell subsets, reflecting not only different maturation stages, but also (at least some degree) of tissue specificity [243].

## Data Availability

Not applicable.
